# Impact of Human Papillomavirus Vaccination, Rwanda and Bhutan

**DOI:** 10.3201/eid2701.191364

**Published:** 2021-01

**Authors:** Iacopo Baussano, Felix Sayinzoga, Ugyen Tshomo, Vanessa Tenet, Alex Vorsters, Daniëlle A.M. Heideman, Tarik Gheit, Massimo Tommasino, Marie Chantal Umulisa, Silvia Franceschi, Gary M. Clifford

**Affiliations:** International Agency for Research on Cancer, Lyon, France (I. Baussano, V. Tenet, T. Gheit, M. Tommasino, M.C. Umulisa, G.M. Clifford);; Ministry of Health, Kigali, Rwanda (F. Sayinzoga);; Jigme Dorji Wangchuck National Referral Hospital, Thimphu, Bhutan (U. Tshomo);; University of Antwerp, Antwerp, Belgium (A. Vorsters);; Vrije Universiteit Amsterdam, Amsterdam, the Netherlands (D.A.M. Heideman);; Centro di Riferimento Oncologico, Aviano, Italy (S. Franceschi)

**Keywords:** papillomavirus infections, papillomavirus vaccines, human papillomavirus recombinant vaccine quadrivalent, type 6, type 11, type 16, type 18, epidemiologic monitoring, disease control, low-income population, viruses, Rwanda, Bhutan, vaccines, vaccine-preventable diseases

## Abstract

Rwanda and Bhutan, 2 low- and middle-income countries, implemented primarily school-based national human papillomavirus (HPV) vaccination in 2011 (Rwanda) and 2010 (Bhutan). We estimated vaccination effectiveness through urine-based HPV prevalence surveys in schools in 2013–2014 and 2017. In Rwanda, 912 participants from baseline surveys and 1,087 from repeat surveys were included, and in Bhutan, 973 participants from baseline surveys and 909 from repeat surveys were included. The overall effectiveness against vaccine-targeted HPV types (i.e., HPV-6/11/16/18) was 78% (95% CI 51%–90%) in Rwanda, and 88% (6%–99%) in Bhutan and against other α-9 types was 58% (21–78) in Rwanda and 63% (27–82) in Bhutan. No effect against other HPV types was detectable. Prevalence of vaccine-targeted HPV types decreased significantly, as well as that of other α-9 types, suggesting cross-protection. These findings provide direct evidence from low- and middle-income countries of the marked effectiveness of high-coverage school-based, national HPV vaccination programs.

Recent estimates suggest that, in the year 2018, ≈570,000 new cervical cancers cases occurred worldwide (*1*). Nearly half of the cases were diagnosed in women <50 years of age, and more than two thirds occurred in low- and middle-income countries (LMICs), particularly in southeastern Asia, Latin America, and sub-Saharan Africa. Human papillomavirus (HPV) types 16 and 18 are responsible for ≈70% of cervical cancers and HPV types 31/33/45/52/58 for another 20% (*2*).

Because cervical cancer is largely preventable, the Director-General of the World Health Organization recently made a global call for action toward the elimination of cervical cancer as a public health problem (*3*). Global implementation of vaccination against HPV with a high coverage underpins the global strategy devised to achieve this ambitious goal (*4*).

Licensed prophylactic HPV vaccines have demonstrated high safety (*5*) and efficacy against persistent HPV infections and precancerous lesions (*6*), and invasive cervical cancers (*7*), and HPV vaccination programs have been shown to be cost-effective in a wide range of settings worldwide (*8*). Furthermore, population-level impact against HPV prevalence and precancerous lesions has been consistently shown in high-income countries (HICs) with well-established HPV national vaccination programs (*9*). HPV vaccine has been disproportionately introduced in high-resource settings, and access to HPV vaccination in LMICs, particularly in Africa and Asia, remains limited (*10*).

Rwanda and Bhutan, both of which are LMICs, started national HPV vaccination programs in 2011 (Rwanda) and 2010 (Bhutan) (Figure 1). Both programs are primarily school-based, introduced a 3-dose schedule of quadrivalent vaccine targeting HPV-6/11/16/18, and switched to a 2-dose schedule in 2015 (Rwanda) and 2016 (Bhutan). In both countries, 12-year-old girls are the target age group for routine vaccination, but both countries had an initial expanded 3-dose catch-up campaign. In Rwanda, since 2011, the national vaccination program targeted all girls attending primary school grade 6 and, in years 2012 and 2013, also targeted girls attending secondary school grade 3, achieving reported coverage of 93% (*11*). In Bhutan, a 1-round catch-up campaign was conducted in 2010, targeting girls 13–18 years of age, achieving reported coverage of 89% (*12*).

**Figure 1:**
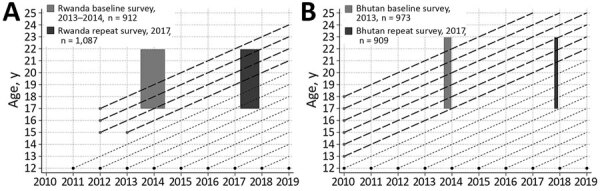
Timing of school-based human papillomavirus vaccination program and surveys in Rwanda (A) and Bhutan (B). Short dashed line represents routine vaccination. Long dashed line represents catch-up vaccination.

The International Agency for Research on Cancer (IARC), in collaboration with the ministries of health of both countries, is conducting long-term studies, including a series of urine surveys, to provide direct evaluation of the population-level impact of HPV vaccination in LMICs (*13*). In 2016, we published the results of the baseline urine surveys among high-school female students (*13*). In this article, we quantify HPV prevalence in repeated surveys, comparing it with the baseline HPV prevalence to estimate population-level impact of HPV vaccination in both countries (*13*).

## Methods

To assess the impact of catch-up HPV vaccination programs in Rwanda and Bhutan, we compared HPV prevalence in women 17–22 years of age in successive urine-based surveys conducted in high schools during 2013–2014 (baseline survey) and 2017 (repeat survey) in both countries (Figure 1). In both countries, the nationwide HPV vaccination program had been launched before the implementation of the baseline surveys. The methods used in the baseline surveys to recruit the study population, collect the urine, extract DNA, and to test and genotype HPV are reported elsewhere (*13*). To ensure comparability of prevalence estimates over time, we used the same methods for the repeat surveys.

### Study Population

In the repeat surveys, we aimed at recruiting ≈1,000 female students 18–20 years of age in each country from the same high schools included in the baseline survey. In Rwanda, we included secondary schools in the Nyarugenge District of Kigali. Of the 22 schools (8 public and 14 private), all but 1 overlapped with the 21 schools included in the baseline survey. In Bhutan, we included high schools in the capital of the country, Thimphu (n = 7), and in the nearby town of Paro (n = 3). Of these 10 schools (3 public and 7 private), 6 overlapped with the 6 schools included in the baseline survey. School authorities gave full support to the conduct of the study, and no school refused participation. The repeat surveys were performed during March–November 2017 (Rwanda) and September–November 2017 (Bhutan). Students in the targeted age groups were invited by school staff to attend study information and recruitment meetings. The large majority of students present at information and recruitment meetings signed the informed consent form, but exact denominators of students by age in each school were not available. In Rwanda, 50 students 17 years of age and 24 students 21 years of age also attended recruitment meetings and were allowed to join the study. Similarly, in Bhutan, 10 students 17 years of age, 68 students 21 years of age, and 30 students 22 years of age also joined the study.

All students who signed an informed consent form received a device for self-collection of urine. Participants were asked to collect first-void urine from the first urination of the day and to return the urine sample on the same morning as collection. Urine samples were recovered at school entry the day after recruitment, and on this day, a short online questionnaire was filled in by a study interviewer (Rwanda) or directly by the student (Bhutan). The questionnaire included information on places of birth and living, history of sexual intercourse, and recalled HPV vaccination status. Urine samples and questionnaires could only be matched through an anonymized identification number.

### Urine Collection and DNA Extraction

Urine samples were self-collected by participants using a device (Colli PeeTM, Novosanis, https://novosanis.com) designed to collect the first 14 mL of first-void urine immediately into 7 mL of a urine-conservation medium to avoid DNA degradation (*14*) and to allow subsequent urine volume to exit the device into the toilet. Self-collected urine samples were gathered and stored on ice at the school on the morning of sample taking; on the same day, the samples were transported to the central laboratory and stored at –20°C until shipment to IARC in cold boxes with ice packs. Subsequently, samples were shipped on dry ice to the Centre for the Evaluation of Vaccination, University of Antwerp, Belgium, where DNA extraction was performed as described elsewhere (*14*). DNA extracts were then shipped back to IARC on dry ice.

### HPV Testing and Genotyping

As in the baseline surveys, 2 methods of different analytical sensitivity were used for HPV testing to overcome the possible problem of the relative lack of sensitivity of HPV detection in urine. The primary HPV testing protocol was performed in the pathology department of Amsterdam University Medical Center, Vrije Universiteit Amsterdam, the Netherlands, where β-globin PCR analysis was first conducted to confirm the presence of human DNA in all specimens (*15*) and a general primer GP5+/6+-mediated PCR with enzyme immunoassay and subsequent genotyping readout was used to detect HPV DNA (*16*). A secondary type-specific E7 PCR bead-based multiplex genotyping assay (E7-MPG) with β-globin primers included was performed at IARC, Lyon, France, using a Luminex bead-based platform (*17*,*18*). This assay also detects DNA from *Chlamydia trachomatis*. Results were considered invalid when β-globin was undetectable by either 1 or both HPV assays.

### Statistical Analyses

**Figure 2:**
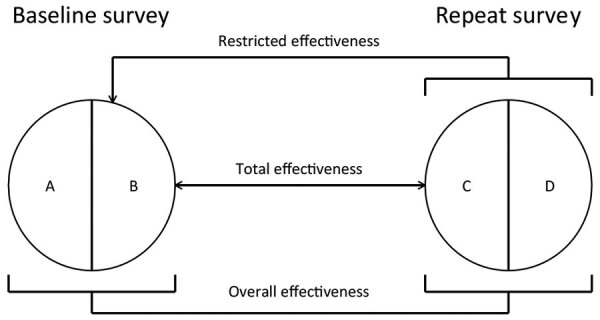
Analytical framework used to assess the impact of human papillomavirus (HPV) vaccination in Rwanda and Bhutan. A) Vaccinated participants in the baseline survey. B) Unvaccinated participants in the baseline survey. C) Vaccinated participants in the repeat survey. D) Unvaccinated participants in the repeat survey. Vaccine effectiveness (VE) was calculated as VE = (1 – PR)%, where PR is a prevalence ratio (PR). Each type of VE is defined according to specific criteria for selecting comparison groups on the basis of reported vaccination status. Overall effectiveness estimates, providing a measure of HPV prevalence reduction over time attributable to vaccination irrespective of the reported vaccination status of each person, were obtained by comparing the type-specific HPV prevalence in all women, unvaccinated and vaccinated, recruited in the baseline and repeat surveys. PR (C and D) / PR (A and B) = overall PR. Restricted effectiveness estimates, providing an approximate estimate of the impact of HPV vaccination versus an entirely unvaccinated population, were obtained by comparing the type-specific HPV prevalence in unvaccinated women in the baseline and all women in repeat surveys. PR (C and D) / PR (B) = restricted PR. Total effectiveness estimates, providing a vaccine efficacy estimate (similar to measures from clinical trials) from real-life settings, were obtained by comparing the type-specific HPV prevalence in unvaccinated women in the baseline and vaccinated women in repeat surveys. PR (C) / PR (B) = total PR, where PR (•) is the type-specific HPV prevalence in each participant group.

For both countries, we compared the distribution of selected characteristics of female students in the baseline and repeat surveys by using χ^2^ tests and a p value of <0.05 for statistical significance. To estimate type-specific HPV prevalence among women recruited in the baseline and repeat surveys, HPV types were grouped as follows: HPV vaccine types (HPV-6, -11, -16, and -18), other α-9 types (HPV-31, -33, -35, -52, and -58), other α-7 types (HPV-39, -45, -59, and -68), and non–α 7/9 types detected by both genotyping tests (HPV-26, -51, -53, -56, -66, -70, -73, and -82). We adapted the framework proposed by Halloran et al. (*19*) to estimate the population-level impact of HPV vaccination in both countries by using different definitions of effectiveness on the basis of increasingly specific criteria to select comparison groups by reported vaccination status (Figure 2). Hence, we compared the type-specific HPV prevalence in all women, unvaccinated and vaccinated, recruited in the baseline and repeat surveys, to compute the overall effectiveness, which provides a measure of HPV prevalence reduction over time attributable to vaccination, irrespective of the reported vaccination status of each person. We also compared the type-specific HPV prevalence in unvaccinated women in the baseline and all women in repeat surveys, to compute the restricted effectiveness to account for the fact that HPV vaccination had already been introduced in both countries when baseline surveys were conducted. Finally, we compared the type-specific HPV prevalence in unvaccinated women in the baseline and vaccinated women in repeat surveys, to compute the total effectiveness, which provides vaccine efficacy estimates from real-life settings (similar to measures from clinical trials).

We computed prevalence ratios (PR) for HPV detection and corresponding 95% CIs by using binomial regression models with a log link. Estimates for Rwanda were adjusted for age group, place of birth, and reported history of sexual intercourse (never vs. ever or prefer not to answer). Estimates for Bhutan were adjusted only for reported history of sexual intercourse because of the small number of infections with HPV vaccine–targeted types observed. HPV vaccine effectiveness (VE) estimates and the corresponding 95% CIs were computed as (1 – PR)%. All statistical analyses were performed by using Stata SE 15.1 (StataCorp, https://www.stata.com).

### Ethics Approval

The research ethics boards of the ministries of health of Rwanda and Bhutan approved the studies in each country. The IARC Ethics Committee approved the studies in both countries.

## Results

In the repeat surveys, 1,198 students in Rwanda and 987 students in Bhutan signed the informed consent forms. Urine samples were not returned for 43 students in Rwanda and 4 students in Bhutan, results were invalid for 38 samples in Rwanda and 49 samples in Bhutan, and 2 additional exclusions were attributable to insufficient DNA for the second test (E7-MPG) in Rwanda. Other participants (28 from Rwanda, 25 from Bhutan) were excluded because of a lack of a questionnaire or because students could not recall their HPV vaccination status. Data from 1,087 students in Rwanda (median age 19 years; range 17–21 years) and 909 students in Bhutan (median age 19 years; range 17–22 years) were included in the final analyses (Table 1; Appendix Figure).

**Table 1:** Comparison of female students in HPV surveys, by selected characteristics, Rwanda baseline (2013–2014) and repeat (2017) surveys and Bhutan baseline (2013) and repeat (2017) surveys*

Characteristic	Rwanda			Bhutan	
	Baseline survey	Repeat survey		Baseline survey	Repeat survey
All	912	1,087		973	909
Age-group, y					
17–18	374 (41.0)	536 (49.3)		285 (29.3)	347 (38.2)
19	274 (30.0)	326 (30.0)		337 (34.6)	303 (33.3)
20–22	264 (29.0)	225 (20.7)		351 (36.1)	259 (28.5)
χ^2^	p<0.001			p<0.001	
Place of birth					
Capital	497 (54.5)	800 (73.6)		309 (31.8)	315 (34.7)
Outside capital	415 (45.5)	287 (26.4)		663 (68.2)	594 (65.3)
χ^2^	p<0.001			p = 0.187	
Place of living					
With family or relative	763 (83.7)	936 (86.1)		798 (82.0)	765 (84.2)
Boarding school	149 (16.3)	151 (13.9)		175 (18.0)	144 (15.8)
χ^2^	p = 0.127			p = 0.215	
History of sexual intercourse					
Never	720 (79.0)	729 (67.1)		871 (89.5)	760 (83.6)
Ever or prefer not to answer†	192 (21.0)	358 (32.9)		102 (10.5)	149 (16.4)
χ^2^	p<0.001			p<0.001	
*Chlamydia trachomatis*‡					
Negative	892 (97.8)	1047 (96.3)		940 (96.6)	872 (95.9)
Positive	20 (2.2)	40 (3.7)		33 (3.4)	37 (4.1)
χ^2^	p = 0.052			p = 0.437	
HPV vaccination					
No	519 (56.9)	125 (11.5)		77 (7.9)	45 (5.0)
Yes	393 (43.1)	962 (88.5)		896 (92.1)	864 (95.0)
χ^2^	p<0.001			p = 0.009	
Age at vaccination§					
<14	12 (3.1)	412 (46.5)		12 (2.0)	569 (87.4)
>14	378 (96.9)	474 (53.5)		591 (98.0)	82 (12.6)
χ^2^	p<0.001			p<0.001	
No. doses§					
1	NA	52 (5.5)		NA	84 (9.9)
2–3	NA	901 (94.5)		NA	769 (90.2)

*Values are no. (%) except as indicated. HPV, human papillomavirus; NA, not assessed. †Includes 4 (Rwanda baseline), and 38 (Rwanda repeat), 43 (Bhutan baseline), and 20 (Bhutan repeat) students who preferred not to answer this question. ‡Detected by using E7 PCR bead-based multiplex genotyping assay. §Does not add up to the total because of missing values.

HPV vaccination was reported by 962 (89%) of study participants in Rwanda and 864 (95%) in Bhutan, and median age at vaccination was 14 years (range 10–18 years) in Rwanda and 12 years (range 10–19 years) in Bhutan. Among vaccinated girls in the studies, 94% in Rwanda and 90% in Bhutan reported being administered >1 dose of vaccine. In the baseline survey, 43% of participants in Rwanda and 92% of participants in Bhutan reported to be vaccinated. In both countries, the distribution of age at vaccination significantly shifted toward younger ages in the repeat surveys compared with baseline surveys. We compared the distribution of participants in the repeat survey by age group and other selected characteristics with the distribution of the same characteristics as observed in the 912 participants in Rwanda and 973 participants in Bhutan in the baseline surveys (Table 1). Students in the repeat surveys were younger than in the baseline surveys and more likely to report sexual intercourse history (33% vs. 21% [p<0.001] in Rwanda and 16% vs. 11% [p<0.001] in Bhutan). In Rwanda, participants enrolled in the repeat survey were also more likely to be born in the capital (Kigali) than in the baseline survey (74% vs. 55% [p<0.001]) and had a higher probability of *Chlamydia trachomatis* detection (4% vs. 2% [p = 0.052]). In both surveys and in both countries, detection of *C. trachomatis* was substantially higher in participants who reported a history of sexual activity (Appendix Table 1).

The distribution of participants’ characteristics by vaccination history is detailed in Appendix Table 2. In the repeat surveys, the distribution of key characteristics was similar between vaccinated and unvaccinated participants in both countries.

**Figure 3:**
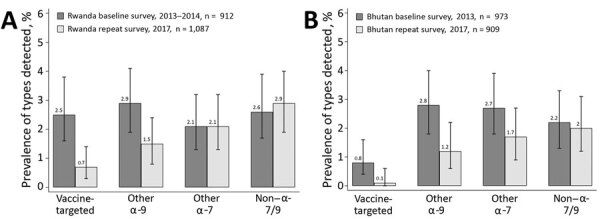
Overall crude human papillomavirus prevalence by general primer GP5+/6+-mediated PCR in baseline and repeat surveys in Rwanda (A) and Bhutan (B), with corresponding 95% CIs. Vaccine-targeted types (HPV-6, -11, -16, -18); other α-9 types (HPV-31, -33, -35, -52, -58); other α-7 types (HPV-39, -45, -59, -68); non–α 7/9 types (HPV-26, -51, -53, -56, -66, -70, -73, -82).

We calculated prevalence and crude PR for groups of HPV types according to GP5+/6+ PCR, in both the baseline and repeat surveys in Rwanda (Figure 3, panel A) and Bhutan (Figure 3, panel B). In Rwanda, the prevalence of vaccine-targeted types decreased 2.5% to 0.7% (crude PR 0.29 [95% CI 0.13–0.65]) and prevalence of other α-9 types decreased from 2.9% to 1.5% (PR 0.52 [95% CI 0.28–0.96]), whereas the prevalence of other α-7 types was 2.1% in both surveys (PR 1.02 [95% CI 0.56–1.85]) and the prevalence of non–α 7/9 HPV types did not significantly change, increasing from 2.6% to 2.9% (PR 1.08 [95% CI 0.64–1.83]). In Bhutan, the prevalence of vaccine-targeted types decreased from 0.8% to 0.1% (PR 0.13 [95% CI 0.02–1.07]), prevalence of other α-9 types decreased from 2.8% to 1.2% (PR 0.44 [95% CI 0.22–0.87]), and prevalence of other α-7 types decreased from 2.7% to 1.7% (PR 0.62 [95% CI 0.33–1.16]), whereas the prevalence of the non–α 7/9 types did not significantly change, decreasing from 2.2% to 2.0% (PR 0.92 [95% CI 0.49–1.71]).

We calculated the adjusted vaccine impact on groups of HPV types in both Rwanda and Bhutan, as measured by estimates of overall, restricted, and total VE (Table 2). In both countries, the precision of statistically significant crude effectiveness estimates improved with adjustment (data not shown, but crude PRs and VE can be calculated from data in Table 2). Overall effectiveness against vaccine-targeted types was 78% (95% CI 51%–90%) in Rwanda and 88% (95% CI 6%–99%) in Bhutan, and increased moving through the scenarios of restricted effectiveness at 86% (95% CI 69%–94%) in Rwanda and 96% (95% CI 52%–100%) in Bhutan, up to a total effectiveness of 95% (95% CI 83%–99%) in Rwanda and 95% (95% CI 49%–100%) in Bhutan. The overall effectiveness against other α-9 types was 58% (95% CI 21%–78%) in Rwanda and 63% (95% CI 27%–82%) in Bhutan, the restricted effectiveness was 63% (95% CI 26%–81%) in Rwanda and 56% (95% CI –89%–90%) in Bhutan, and the total effectiveness was 60% (95% CI 19%–81%) in Rwanda and 58% (95% CI –81%–90%). In neither country were any effectiveness estimates against other HPV α-7 or non–α 7/9 types ever statistically significant.

**Table 2:** PRs and VE for positivity for human papillomavirus by GP5+/6+ PCR, Rwanda and Bhutan*

Country and type of effectiveness	HPV type†	No. (%) by vaccination status		Adjusted PRs (95% CI)‡	Adjusted VE, % (95% CI)‡
		Baseline survey	Repeat survey		
Rwanda					
Overall§		All	All		
	No.	912	1,087		
	Vaccine-targeted	23 (2.5)	8 (0.7)	0.22 (0.10 to 0.49)	78 (51 to 90)
	Other α-9	26 (2.9)	16 (1.5)	0.42 (0.22 to 0.79)	58 (21 to 78)
	Other α-7	19 (2.1)	23 (2.1)	0.82 (0.44 to 1.52)	18 (–52 to 56)
	Non–α 7/9	24 (2.6)	31 (2.9)	0.85 (0.50 to 1.45)	15 (–45 to 50)
Restricted¶		Unvaccinated	All		
	No.	519	1,087		
	Vaccine-targeted	21 (4.0)	8 (0.7)	0.14 (0.06 to 0.31)	86 (69 to 94)
	Other α-9	17 (3.3)	16 (1.5)	0.37 (0.19 to 0.74)	63 (26 to 81)
	Other α-7	12 (2.3)	23 (2.1)	0.71 (0.35 to 1.43)	29 (–43 to 65)
	Non–α 7/9	12 (2.3)	31 (2.9)	0.95 (0.49 to 1.85)	5 (–85 to 51)
Total#		Unvaccinated	Vaccinated		
	No.	519	962		
	Vaccine-targeted	21 (4.0)	3 (0.3)	0.05 (0.01 to 0.17)	95 (83 to 99)
	Other α-9	17 (3.3)	15 (1.6)	0.40 (0.19 to 0.81)	60 (19 to 81)
	Other α-7	12 (2.3)	19 (2.0)	0.65 (0.31 to 1.37)	35 (–37 to 69)
	Non–α 7/9	12 (2.3)	25 (2.6)	0.86 (0.43 to 1.71)	14 (–71 to 57)
Bhutan					
Overall§		All	All		
	No.	973	909		
	Vaccine-targeted	8 (0.8)	1 (0.1)	0.12 (0.01 to 0.94)	88 (6 to 99)
	Other α-9	27 (2.8)	11 (1.2)	0.37 (0.18 to 0.73)	63 (27 to 82)
	Other α-7	26 (2.7)	15 (1.7)	0.49 (0.26 to 0.92)	51 (8 to 74)
	Non–α 7/9	21 (2.2)	18 (2.0)	0.77 (0.41 to 1.42)	23 (–42 to 59)
Restricted¶		Unvaccinated	All		
	No.	77	909		
	Vaccine-targeted	2 (2.6)	1 (0.1)	0.04 (0 to 0.48)	96 (52 to 100)
	Other α-9	2 (2.6)	11 (1.2)	0.44 (0.10 to 1.89)	56 (–89 to 90)
	Other α-7	1 (1.3)	15 (1.7)	1.08 (0.15 to 7.82)	–8 (–682 to 85)
	Non–α 7/9	3 (3.9)	18 (2.0)	0.47 (0.14 to 1.59)	53 (–59 to 86)
Total#		Unvaccinated	Vaccinated		
	No.	77	864		
	Vaccine-targeted	2 (2.6)	1 (0.1)	0.05 (0 to 0.51)	95 (49 to 100)
	Other α-9	2 (2.6)	10 (1.2)	0.42 (0.10 to 1.81)	58 (–81 to 90)
	Other α-7	1 (1.3)	15 (1.7)	1.13 (0.16 to 8.21)	–13 (–721 to 84)
	Non–α 7/9	3 (3.9)	17 (2.0)	0.46 (0.15 to 1.48)	54 (–48 to 85)

*PR, prevalence ratio; VE, vaccine effectiveness. †Vaccine-targeted types (HPV-6, -11, -16, -18); other α-9 types (HPV-31, -33, -35, -52, -58); other α-7 types (HPV-39, -45, -59, -68); non–α 7/9 types (HPV-26, -51, -53, -56, -66, -70, -73, -82). ‡Adjusted for age, ever had sexual intercourse, and place of birth in Rwanda, and for ever had sexual intercourse only in Bhutan. §Entire baseline group compared with entire repeat group. ¶Unvaccinated baseline group compared with entire repeat group. #Unvaccinated baseline group compared with vaccinated repeat group.

According to testing with the more sensitive E7-MPG protocol, all HPV prevalence estimates were consistently higher, and corresponding HPV VE estimates consistently lower than the corresponding estimate shown for GP5+/6+. Also, in Bhutan, restricted and total effectiveness were not statistically significant in the E7-MPG PCR (Appendix Table 3).

## Discussion

By comparing type-specific HPV prevalence among young women in repeat surveys, we have assessed the early impact of HPV vaccination at the population-level in Rwanda and Bhutan, 2 LMICs implementing a national HPV vaccination program. In both countries, high-coverage in schools (≈90%) with quadrivalent vaccine has vastly decreased the prevalence of HPV types targeted by the vaccine (HPV-6, -11, -16, and -18), as well as significantly decreasing also that of other α-9 HPV types (HPV-31, -33, -35, -52, and -58), suggesting cross-protection (58% in Rwanda and 63% in Bhutan). On the other hand, no changes were observed in other HPV types during this period, suggesting that the prevalence reduction observed in both countries is entirely vaccine-driven and not attributable to changes over time in sexual behavior.

An important strength of our present study is the comparability of HPV prevalence estimates in the baseline and repeat surveys in both countries. To this end, we adopted the same methods and procedures to recruit, interview, and test young women and used, as far as feasible, the same high-schools to recruit study participants. To account for behavioral changes that might have occurred in the source population, we adjusted our estimates for the reported history of sexual intercourse, which was more frequent in repeat surveys in both countries. The prevalence of non–α 7/9 HPV types (for which no prior evidence for cross-protection exists) did not significantly change over time. We also did not observe any indications of type replacement.

In both countries, HPV vaccination had been introduced before the implementation of the baseline surveys; 43% of study participants in Rwanda and 92% in Bhutan were vaccinated in the catch-up campaigns. Hence, our effectiveness estimates are underestimated because of vaccine-induced protection in the reference group. In particular, estimates of overall effectiveness are affected by both direct and indirect protection in the baseline group, whereas restricted and total effectiveness estimates are affected only by indirect protection. Furthermore, some baseline survey participants might have been sexually active and HPV-infected before being vaccinated. In Bhutan, because of the high vaccination coverage, the number of participants positive to vaccine-targeted HPV types in the reference group was tiny; therefore, effectiveness estimates are imprecise. Nevertheless, irrespective of the genotyping method considered, overall effectiveness was >80%. Restricted and total effectiveness were not statistically significant in tests using E7-MPG PCR. By contrast, in Rwanda where vaccination coverage in the reference group was much lower, estimates of effectiveness against vaccine-targeted and other α-9 types are more precise and consistently statistically significant.

We used 2 HPV testing methods of different analytical sensitivity (GP5+/6+ PCR and E7-MPG) to enable us to compare possibly different estimates of VE by assay and overcome the possible problem of the relative lack of sensitivity of HPV detection in urine. Significant overall effectiveness was shown with both methods used. However, we estimated stronger overall VE (78% in Rwanda and 88% in Bhutan) when HPV was measured by using GP5+/6+ PCR. The lower estimated VE might relate to the increased detection of low-level HPV DNA by E7-MPG that might have no clinical significance (*20*). In Bhutan, as mentioned previously, restricted and total effectiveness estimates were not statistically significant most likely because of few HPV vaccine-type positive women.

Population-level impact of HPV vaccination with both bivalent and quadrivalent vaccine, as well as cross-protection against other high-risk HPV types, have been repeatedly documented in HICs (*21*) The magnitude of the reduction in prevalence of cervical HPV types targeted by the quadrivalent vaccine impact estimated in Rwanda and Bhutan is similar to that recently recorded in repeat cross-sectional studies conducted in Australia (93% among women <25 years of age) (*22*) and the United States (86% among 14- to 19-year-olds and 71% among 20- to 24-year-olds) (*23*). The size of cross-protection of quadrivalent vaccine against α-9 HPV types estimated in our study is consistent with estimates reported in Australia (60% against HPV-31, -33, and -45) (*24*), the post-hoc analysis of trial data (22% against HPV-31, -33, -35, -52, and -58) (*25*), and findings of a metaanalysis summarizing data from HICs (17% against HPV-31, -33, -35, -45, -52, and -58) (*21*).

The most relevant limitation of our study is that baseline surveys could not be conducted in unvaccinated populations. As a result, overall effectiveness estimates are likely to be underestimated in both countries and the statistical power of the study, in particular in Bhutan, is reduced. For Bhutan, the overall effectiveness against vaccine-targeted HPV types reported in this article is the same as that estimated from surveys based on cervical cell samples (88% [95% CI 80%–92%]) (*26*). Furthermore, vaccination status was self-reported and could not be ascertained by using vaccination registries. This limitation might have particular impact on the estimations of restricted and total effectiveness, which use HPV prevalence among unvaccinated participants in baseline surveys as a reference category. To minimize the risk for recall bias, questions about HPV vaccination were accompanied by a detailed description of the vaccination process as implemented in each country. Furthermore, no other vaccine was given to age-groups targeted with HPV vaccination in the same period. Finally, our surveys were not designed, nor powered, to disentangle direct and indirect (herd) vaccine-induced protection, which has been repeatedly observed in studies conducted in HICs (*9*,*24*,*27*,*28*). However, the high overall effectiveness estimated in Bhutan (88%), despite very similar HPV vaccination coverage in the 2 surveys, suggests vaccine-induced indirect herd effect provided by older birth cohorts to younger ones. In the surveys based on cervical cell samples conducted in Bhutan, the indirect effectiveness was 78% (95% CI 61%–88%) (*26*). Such an intercohort protection mechanism has been observed in other community-randomized trials (*29*) and elucidated through modeling studies (*30*).

In summary, our study provides direct evidence from LMICs of the marked effectiveness of a high-coverage national catch-up HPV vaccination program. The full impact of vaccination of the routine target cohorts, vaccinated before sexual debut, remains to be measured; continued monitoring will be necessary to assess the sustained impact of the 2-dose HPV vaccination schedule recently introduced in both countries. The reported findings will also be instrumental in supporting a long-term stakeholders’ commitment to HPV vaccination, to inform future budget allocation exercises, and to adapt local cervical cancer screening programs to vaccinated populations. Furthermore, the repeat urine-based survey approach used in Rwanda and Bhutan to monitor the impact of HPV vaccination is well-accepted and remarkably adaptable to a wide range of settings and populations, making this approach particularly valuable to periodically assess the early impact of HPV vaccination.

Appendix Additional information about impact of human papillomavirus vaccination, Rwanda and Bhutan.
