# Supplementary material for: Impact of Human Papillomavirus Vaccination, Rwanda and Bhutan
Source: Emerg Infect Dis. 2021 Jan;27(1):1–9. doi: 10.3201/eid2701.191364 (PMC7774553; doi:10.3201/eid2701.191364)
Supplement: Appendix — Additional information about impact of human papillomavirus vaccination, Rwanda and Bhutan. [file 19-1364-Techapp-s1.pdf]

Article DOI: https://doi.org/10.3201/eid2701.191364

Impact of Human Papillomavirus Vaccination, Rwanda and Bhutan

Appendix

Appendix Table 1. Chlamydia trachomatis detection*, by reported sexual history in baseline and repeat surveys. Rwanda baseline (2013–14) and repeat (2017) surveys, and Bhutan baseline (2013) and repeat (2017) surveys

	Rwanda				Bhutan			
	Baseline survey		Repeat survey		Baseline survey		Repeat survey	
Characteristic	No.	No. detected (%)	No.	No. detected (%)	No.	No. detected (%)	No.	No. detected (%)
All	912	20 (2.2)	1,087	40 (3.7)	973	33 (3.4)	909	37 (4.1)
Sexually not active	720	8 (1.1)	729	13 (1.8)	871	24 (2.8)	760	18 (2.4)
Sexually active	192	12 (6.3)	358	27 (7.5)	102	9 (8.8)	149	19 (12.8)

*Detected by E7-MPG.

Appendix Table 2. Comparison of HPV-vaccinated and unvaccinated female students by selected characteristics. Rwanda baseline (2013-14) and repeat (2017) surveys and Bhutan baseline (2013) and repeat (2017) surveys

	Rwanda				Bhutan			
	Baseline survey		Repeat survey		Baseline survey		Repeat survey	
	Vaccinated	Unvaccinated	Vaccinated	Unvaccinated	Vaccinated	Unvaccinated	Vaccinated	Unvaccinated
Characteristic	No. (%)	No. (%)	No. (%)	No. (%)	No. (%)	No. (%)	No. (%)	No. (%)
All	393	519	962	125	896	77	864	45
Age-group (y)								
17–18	211 (53.7)	163 (31.4)	488 (50.7)	48 (38.4)	275 (30.7)	10 (13.0)	330 (38.2)	17 (37.8)
19	82 (20.9)	192 (37.0)	285 (29.6)	41 (32.8)	319 (35.6)	18 (23.4)	291 (33.7)	12 (26.7)
20–22	100 (25.5)	164 (31.6)	189 (19.7)	36 (28.8)	302 (33.7)	49 (63.6)	243 (28.1)	16 (35.6)
χ^2	$p < 0.001$		$p = 0.016$		$p < 0.001$		$p = 0.481$	
Place of birth								
Capital	198 (50.4)	299 (57.6)	716 (74.4)	84 (67.2)	293 (32.7)	16 (20.8)	303 (35.1)	12 (26.7)
Outside capital	195 (49.6)	220 (42.4)	246 (25.6)	41 (32.8)	602 (67.3)	61 (79.2)	561 (64.9)	33 (73.3)
χ^2	$p = 0.030$		$p = 0.085$		$p = 0.031$		$p = 0.248$	
Place of living								
With family/relative	336 (85.5)	427 (82.3)	826 (85.9)	110 (88.0)	733 (81.8)	65 (84.4)	725 (83.9)	40 (88.9)
Boarding school	57 (14.5)	92 (17.7)	136 (14.1)	15 (12.0)	163 (18.2)	12 (15.6)	139 (16.1)	5 (11.1)
χ^2	$p = 0.192$		$p = 0.516$		$p = 0.568$		$p = 0.373$	
History of sexual intercourse								
Never	317 (80.7)	403 (77.7)	647 (67.3)	82 (65.6)	804 (89.7)	67 (87.0)	722 (83.6)	38 (84.4)
Ever/Prefer not to answer*	76 (19.3)	116 (22.4)	315 (32.7)	43 (34.4)	92 (10.3)	10 (13.0)	142 (16.4)	7 (15.6)
χ^2	$p = 0.269$		$p = 0.711$		$p = 0.455$		$p = 0.877$	
Chlamydia trachomatis†								
Negative	387 (98.5)	505 (97.3)	928 (96.5)	119 (95.2)	866 (96.7)	74 (96.1)	831 (96.2)	41 (91.1)
Positive	6 (1.5)	14 (2.7)	34 (3.5)	6 (4.8)	30 (3.4)	3 (3.9)	33 (3.8)	4 (8.9)
χ^2	$p = 0.232$		$p = 0.480$		$p = 0.799$		$p = 0.093$	

*Includes 43 (Bhutan baseline) and 20 (Bhutan repeat); 4 (Rwanda baseline) and 38 (Rwanda repeat) students, who preferred not to answer this question.
†Detected by E7-MPG.

Appendix Table 3. Prevalence ratios (PRs) and vaccine effectiveness (VE) with corresponding 95% confidence intervals (CIs) for positivity for human papillomavirus by E7-MPG. Rwanda and Bhutan

Country	Type of effectiveness	HPV type*	No. (%) by vaccination status: Baseline survey	No. (%) by vaccination status: Repeat survey	Adjusted PRs†	Adjusted VE (%)†
Rwanda						
	Overall		All	All		
		No.	912	1,087		
		Vaccine-targeted	44 (4.8)	37 (3.4)	0.63 (0.41 to 0.97)	37 (3 to 59)
		Other α-9	68 (7.5)	67 (6.2)	0.70 (0.51 to 0.97)	30 (3 to 49)
		Other α-7	44 (4.8)	75 (6.9)	1.14 (0.80 to 1.62)	−14 (−62 to 20)
		Non-α 7/9	74 (8.1)	123 (11.3)	1.15 (0.88 to 1.50)	−15 (−50 to 12)
	Restricted		Unvaccinated	All		
		No.	519	1,087		
		Vaccine-targeted	33 (6.4)	37 (3.4)	0.48 (0.30 to 0.77)	52 (23 to 70)
		Other α-9	49 (9.4)	67 (6.2)	0.59 (0.42 to 0.84)	41 (16 to 58)
		Other α-7	27 (5.2)	75 (6.9)	1.06 (0.70 to 1.61)	−6 (−61 to 30)
		Non-α 7/9	48 (9.2)	123 (11.3)	1 (0.73 to 1.37)	0 (−37 to 27)
	Total		Unvaccinated	Vaccinated		
		No.	519	962		
		Vaccine-targeted	33 (6.4)	21 (2.2)	0.29 (0.17 to 0.50)	71 (50 to 83)
		Other α-9	49 (9.4)	55 (5.7)	0.54 (0.37 to 0.78)	46 (22 to 63)
		Other α-7	27 (5.2)	65 (6.8)	1.05 (0.69 to 1.61)	−5 (−61 to 31)
		Non-α 7/9	48 (9.2)	102 (10.6)	0.93 (0.67 to 1.29)	7 (−29 to 33)
Bhutan						
	Overall		All	All		
		No.	973	909		
		Vaccine-targeted	12 (1.2)	2 (0.2)	0.17 (0.04 to 0.75)	83 (25 to 96)
		Other α-9	33 (3.4)	44 (4.8)	1.20 (0.78 to 1.85)	−20 (−85 to 22)
		Other α-7	40 (4.1)	36 (4.0)	0.85 (0.55 to 1.32)	15 (−32 to 45)
		Non-α 7/9	56 (5.8)	62 (6.8)	1.08 (0.76 to 1.52)	−8 (−52 to 24)
	Restricted		Unvaccinated	All		
		No.	77	909		
		Vaccine-targeted	1 (1.3)	2 (0.2)	0.16 (0.01 to 1.77)	84 (−77 to 99)
		Other α-9	2 (2.6)	44 (4.8)	1.76 (0.44 to 6.97)	−76 (−597 to 56)
		Other α-7	2 (2.6)	36 (4.0)	1.42 (0.35 to 5.76)	−42 (−476 to 65)
		Non-α 7/9	7 (9.1)	62 (6.8)	0.69 (0.34 to 1.41)	31 (−41 to 66)
	Total		Unvaccinated	Vaccinated		
		No.	77	864		
		Vaccine-targeted	1 (1.3)	2 (0.2)	0.17 (0.02 to 1.86)	83 (−86 to 98)
		Other α-9	2 (2.6)	42 (4.9)	1.76 (0.45 to 6.97)	−76 (−597 to 55)
		Other α-7	2 (2.6)	33 (3.8)	1.36 (0.34 to 5.51)	−36 (−451 to 66)
		Non-α 7/9	7 (9.1)	58 (6.7)	0.68 (0.33 to 1.38)	32 (−38 to 67)

*Vaccine-targeted types (HPV-6, -11, -16, -18); other α-9 types (HPV-31, -33, -35, -52, -58); other α-7 types (HPV-39, -45, -59, -68); non-α 7/9 types (HPV-26, -51, -53, -56, -66, -70, -73, -82).
†Adjusted for age, ever had sexual intercourse, and place of birth in Rwanda and for ever had sexual intercourse only in Bhutan.

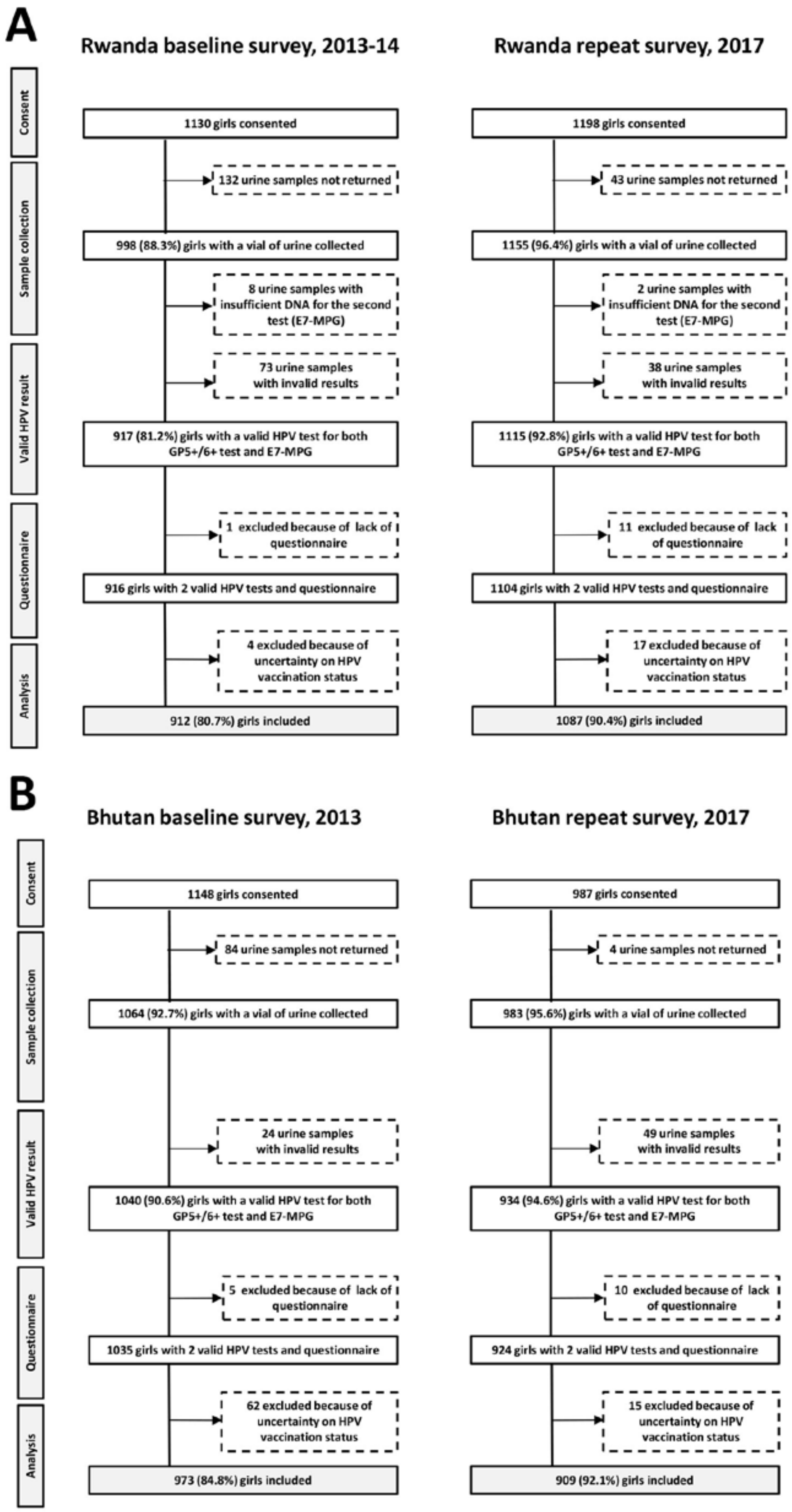

Appendix Figure. Study flow-chart. Rwanda baseline (2013–14) and repeat (2017) surveys, and Bhutan baseline (2013) and repeat (2017) surveys.
